# The action-sentence compatibility effect in third person action sentence comprehension

**DOI:** 10.3389/fpsyg.2025.1562351

**Published:** 2025-06-04

**Authors:** Zhiwei Cai, Bing Qi, Ning Fan

**Affiliations:** ^1^College of Teacher Education, Baoding University, Baoding, Hebei, China; ^2^College of Education, Hebei University, Baoding, Hebei, China

**Keywords:** third person, action-sentence comprehension, ACE, perceptual symbol systems, indexical hypothesis

## Abstract

This study investigates the influence of object animacy and perspective priming on the Action-sentence Compatibility Effect (ACE) in third-person action sentences using the sentence sensibility judgment paradigm. Experiment 1 showed that ACE occurred along the front-back axis when the object was an inanimate noun. Experiment 2 revealed that ACE appeared along the left–right axis when the object was a personal name. Experiment 3 demonstrated that ACE aligned with the action’s direction under agent priming, while ACE shifted to the opposite direction under patient priming. These findings suggest that participants can flexibly simulate the perspective of an action based on spatial when processing third-person action sentences.

## 1 Introduction

Action-sentence Compatibility Effect (ACE) reflects the interaction between language and action, which is essential evidence of embodied cognition (Winter et al., 2022; Zwaan, 2021). The ACE was found when the direction of action described in the sentence read by the participants is consistent with the direction of the arm movement, the arm movement response is promoted and the reaction time is shortened; otherwise, the arm response is interfered and the reaction time is increased (Glenberg and Kaschak, 2002). For example, when participants read a sentence describing an action toward the body, such as “You open a drawer,” their button-press response is faster with a bent arm moving backward than with an arm moving forward.

Although ACE has been observed across different experimental paradigms and languages (Santana and de Vega, 2013; Gianelli et al., 2011; van Dam and Desai, 2017), recent findings suggest its instability, particularly when action sentences use third-person pronouns as subjects. Previous studies have shown that readers tend to simulate actions from the agent’s perspective when processing second-person pronoun subjects, whereas they adopt an observer’s perspective for sentences with third- or first-person pronoun subjects. The preference for action perspective simulation varies among individuals and is not essential for sentence comprehension (Brunyé et al., 2016). However, readers engage in action perspective simulation automatically, even in memory tasks solely related to actions (Ditman et al., 2010). This suggests that when readers process second-person action sentences, they activate richer perceptual and motor representations. Additionally, in ACE studies, action sentences containing second-person pronouns tend to produce more stable ACE. Numerous studies investigating second-person sentences with “you” as the subject or object have found that action sentences involving movements toward or away from the body elicit ACE (Glenberg and Kaschak, 2002; Schwarzkopf et al., 2011).

However, subsequent research has shown that the ACE less consistent when participants process action sentences with third-person subjects. Specifically, ACE appears only in sentences where the object is an inanimate noun (Bergen and Wheeler, 2005) but not when the object is an animate personal name (Gianelli et al., 2011; Li, 2015; Schwarzkopf et al., 2011). Gianelli et al. (2011) found that when participants read action sentences with both the subject and object in the third person, ACE appeared whether they completed the task from the third-person perspective or taken the agent/patient perspective. Therefore, the instability of ACE may arise because, when processing action sentences with third-person subjects and objects, readers do not take the agent/patient perspective, but instead tend to taken the observer perspective.

Unlike the agent/patient perspective, the observer perspective may involve mental simulation of other spatial dimensions. In the agent/patient perspective, actions approaching or moving away from the body align with the front-back response axis, while the observer perspective may correspond to the left–right response axis. This suggests that the mental simulation in the process of language understanding is completed under the guidance of syntax and semantics (Bergen and Chang, 2005) and the animacy of sentence subject (Trueswell et al., 1994) and object (Roehm et al., 2004) can be processed by readers in real-time, furthermore, influence the reader to the sentence’s mental simulation. Research findings show that the ACE disappears when the spatial dimension of mental simulation does not align with the perspective. For example, when readers simulate the agent/patient perspective but the response direction is along the left–right axis, the ACE disappears (Gianelli et al., 2011; van Dam and Desai, 2017). Therefore, sentences with third-person subject and object do not specify whether the reader should adopt the agent/patient perspective, which may lead the readers to take an observer’s perspective. In this case, influenced by the left-to-right orthographic direction of their native language, the subject (agent) is mentally simulated on the left side of space, while the object (patient) is simulated on the right side (Boiteau and Almor, 2017). Specifically, actions approaching the agent are simulated as moving to the left, while actions moving away from the agent are simulated as moving to the right, resulting in ACE along the left–right axis (van Dam and Desai, 2017).

According to Indexical Hypothesis, ACE comes from readers’ mental simulation of verb in sentences (Glenberg and Robertson, 1999, 2000). Simulate the motion of approaching and moving away from the body according to the meaning of the verb. According to Indexical Hypothesis, this absent of ACE can be attributed to the absence of explicit agent/patient roles, which are more readily identifiable in second-person sentences featuring “you.” However, this theory does not account for the appearance of ACE along the left–right axis in sentences involving actions approaching or moving away from the body.

The theory of Perceptual Symbol Systems suggest that readers simulate movements and perceptual experiences, including spatial perception (Barsalou, 1999). Spatial simulation is the basis of action simulation (Beveridge and Pickering, 2013). Reader can adopt either the agent/patient perspective or an observer perspective, and spatial simulation influencing the simulation of approaching and away actions. The mental simulation of ACE is thus an action simulation process embedded within a spatial framework. Additionally, the animacy of object may affect the spatial framework of action simulation, so that the direction of action simulation is not only limited to the direction of verb action, but also can appear in the left–right axis. However, no research has systematically tested this theoretical explanation.

Furthermore, if action simulation is based on spatial simulation, the absence of ACE along the front-back axis in sentences with third-person subject-object sentences (Schwarzkopf et al., 2011; Li, 2015), may be due to the lack of a clear spatial reference frame, causing readers to alternate between simulating themselves as the agent and as the patient perspective. Studies on spatial perspective taking have found that the perspective of individuals in cognitive processing is not fixed and can be converted (Klaus and Anne, 2010). After taking different perspectives, the participants would make spatial perception judgments and false belief judgments from the corresponding perspectives, which had an impact on cognitive processes such as prosocial thinking and trust (Erle and Topolinski, 2017; Erle et al., 2018; Xie et al., 2018). Therefore, sentences with third-person subject and object, which may lead the readers to take an observer’s perspective, causing ACE to appear along the left–right axis. Once the agent/patient perspective is activated, the reader’s perspective becomes clearer, and ACE may appear along the front-back axis. Meanwhile, the agent/patient perspective is influenced by different spatial positions, and ACE may manifest in a direction opposite to the verb. As found by Gianelli et al. (2011) in Experiment 3, when the agent/patient perspective is activated at left or right along the left–right axis, the direction and strength of ACE change. However, some studies have shown that during language comprehension, participants may have a preference for the perspective they taken, tending to choose a specific perspective, such as the agent perspective or the observer perspective (Hartung et al., 2017; Vukovic and Williams, 2015). Therefore, whether spatial perspective can activate the ACE along the front-back axis in third-person subject and object action sentences is also a question of interest in this study.

In summary, this study included three experiments. Experiment 1 used sentences with personal names as subjects and object names as objects, while Experiment 2 used sentences with personal names as both subjects and objects. These experiments explored the influence of object animacy on ACE along both the front-back axis and left–right axis. Experiment 3 tested the spatial simulation based on perceptual symbol system theory, employing a perspective priming paradigm with action sentences where a personal name appeared as both the subject and the object. If the direction of ACE remains unaffected by the animacy of the object, the findings support the index hypothesis. Conversely, if the direction of ACE varies with the animacy and is influenced by perspective priming, the results provide evidence for the theory of perceptual symbolic systems.

## 2 Experiment 1

### 2.1 Participants and materials

G*Power3.1 was used to calculate the required number of participants. Based on a medium effect size (*f* = 0.20), a statistical test power 1-*β* = 0.95, and a significance level of *α* = 0.05, the minimum required sample size was 112. A total of 128 college students (68 females), aged between 19 and 27 years old (*M* = 21.73, *SD* = 2.28), native Chinese, right-handed, normal visual acuity or corrected visual acuity, were recruited. The subjects were randomly assigned equally to each of the four experimental conditions.

This study developed 100 sentences with personal names as subjects and object nouns as the objects, as well as 80 sentences with personal names as both subjects and objects. A group of 31 undergraduate students who did not participate in the main experiment were asked to rate the sentences on several dimensions: directionality (1 = very toward the body, 7 = very away from body), concreteness (1 = very concrete, 7 = very abstract), validity (1 = very unreasonable, 5 = very reasonable), familiarity (1 = very unfamiliar, 5 = very familiar) and emotional valence (1 = very negative, 9 = very positive). A total of 78 sentences with concrete semantics (abstractness ≤ 3), clear direction meaning (sentence describing actions toward the body ≤ 3, sentence describing actions away from body ≥ 5) and reasonable content were selected as candidate materials. From these, 20 sentences with object nouns as the objects were chosen, including 10 sentences describing actions toward the body (e.g., 谢琨**领取**了食物/Xie Kun received the food) and 10 sentences describing action away from the body (e.g., 孙慧**发出**了礼物/Sun Hui sent out the gift). The two groups of sentences were matched for concreteness, validity, familiarity, emotional valence and stroke count using independent sample *t*-test.

To prevent participants from guessing the purpose of the experiment, 20 filler sentences with the subject or object as the second person were created without formal analysis. Additionally, 40 nonsensical sentences (e.g., 你**拿起**了大地/you picked up the earth) were included to balance participants’ responses. Among these, 15 sentences had “你/you” as the subject (e.g., 你**印刷**了石头/you print the stone), 15 sentences have the personal name as the subject (e.g., li Juan drilling tears/李娟钻进了眼泪), and 10 sentences have personal name as both the subject and object (e.g., 张龙**枯萎**了刘丽/Zhang long get withered Liu li).

### 2.2 Design and procedure

The experiment employed a 2 × 2 × 2 design with the following factors: response axis (front-back axis, left–right axis), reasonable response direction (toward the body: back/left; away from the body: front/right), and sentence types (describing action toward the body, describing action away from the body). Response axis and reasonable response direction were between-subject variables, while sentence types were a within-subject variable. The dependent variables were key response time and accuracy in sentence validity judgment.

**Figure 1:**
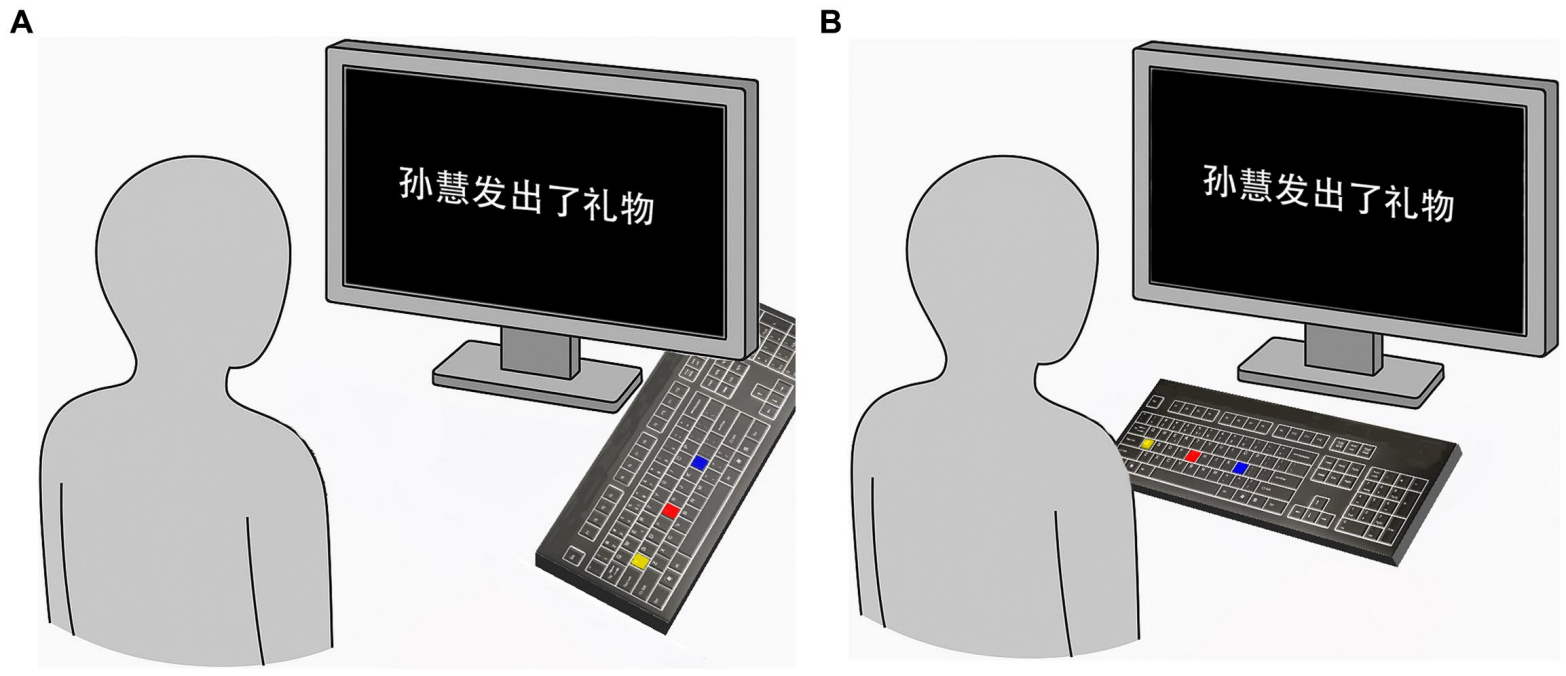
**(A)** Front-back axis condition. **(B)** Left-right axis condition.

The participants used their right index finger to respond on a QWERTY keyboard, with red, yellow, and blue labels attached to the G, A, and L keys, respectively. The three keys were aligned in a straight line, with the red key positioned between the yellow and blue keys, each separated by three keys. The sentence was displayed in Song font at size 30 in the center of the screen, with the participants seated about 50 cm away from the screen. Both the sentence and the keyboard were within the participants’ line of sight, allowing them to read and press the keys without turning their heads or looking down. In the front-back axis condition, the keyboard was placed flat on the desk, rotated 90° counterclockwise to be perpendicular to the participant’s body, with the yellow key positioned closer to the body and the blue key positioned farther from the body (see Figure 1A). In the left–right axis condition, the keyboard remained in its normal orientation, parallel to the participant’s body, with the yellow key on the left and the blue key on the right (see Figure 1B).

The participants were required to make accurate and rapid key responses to determine the validity of the sentences. The experiment was programmed using E-Prime 3.0 software, and each participant randomly read 80 sentences. Each trial began with a 500 ms fixation point (“+ “) followed by the presentation of the sentence after an interval of 300-500 ms. The sentence was displayed for a maximum of 5 s, after which the participant made a key response, causing the sentence to disappear. This was followed by a 1,000 ms blank screen. After practice, participants proceeded to the formal experiment, which lasted 10–15 min, with 10 valid trials for each condition.

### 2.3 Results

Due to the strict matching criteria, the number of sentences used in the experiment was relatively limited. To ensure sufficient data for statistical comparisons, we set 85% as the accuracy threshold. This threshold was determined based on common practice in similar studies (e.g., Diefenbach et al., 2013) and the need to balance data quality with sample retention. Data were analyzed using SPSS 21. Eight participants with accuracy rates lower than 85% were excluded. Trials with incorrect judgments and response times more than 2.5 standard deviations away from the mean in each condition were removed, resulting in a data exclusion rate of 3.25%. Descriptive statistics for mean response times and accuracy rates are presented in Table 1.

**Table 1:** Average reaction times and accuracy of sentences in Experiment 1 (*M* ± *SD*).

Response axis	Reasonable response direction	RT (*ms*)		ACC (*%*)	
		STB	SAB	STB	SAB
Front-back axis	Toward the body (back)	1,201 ± 241	1,403 ± 228	97.56 ± 4.79	95.11 ± 7.62
	Away from the body (front)	1,314 ± 209	1,211 ± 235	98.00 ± 3.57	97.78 ± 4.74
Left–right axis	Toward the body (back)	1,328 ± 279	1,300 ± 279	98.22 ± 3.47	98.67 ± 4.07
	Away from the body (front)	1,240 ± 226	1,259 ± 270	97.33 ± 3.76	95.33 ± 6.35

STB, sentence describing actions toward the body; SAB, sentence describing actions away from the body.

The results of the repeated measures ANOVA on mean response times shown that the main effect of response axis was not significant [*F*_(1, 116)_ < 0.001, *p* = 0.995], the main effect of reasonable response direction was not significant [*F*_(1, 116)_ = 2.815, *p* = 0.096], and the main effect of sentence types was not significant [*F*_(1, 116)_ = 0.483, *p* = 0.488].

The interaction between response axis and reasonable response direction was not significant [*F*_(1, 116)_ = 0.170, *p* = 0.681], the interaction between response axis and sentence types was not significant [*F*_(1, 116)_ = 0.679, *p* = 0.412], and the interaction between reasonable response direction and sentence types was not significant [*F*_(1, 116)_ = 3.859, *p* = 0.052].

However, the three-way interaction between response axis, correct response direction, and sentence type was significant [*F*_(1, 116)_ = 7.241, *p* = 0.008, $\eta_{p}^{2}$ = 0.059]. Analysis of simple main effects showed that, for the front-back axis, when the correct key was pressed backward, the response time for sentence describing actions toward the body is faster compared to those describing actions away from the body (*F*_(1, 116)_ = 9.532, *p* = 0.003, $\eta_{p}^{2}$ = 0.076, 95%CI = [72.28, 330.96]). However, when the correct key was pressed forward, there was no significant difference in response times between sentences describing actions toward the body and those describing actions away from the body [*F*_(1, 116)_ = 2.459, *p* = 0.120]. For the left–right axis, no significant differences in response times were found between sentence types when the correct key was pressed left [*F*_(1, 116)_ = 0.183, *p* = 0.670] or right [*F*_(1, 116)_ = 0.089, *p* = 0.766].

The accuracy analysis results (see Supplementary materials) showed no main effects or interaction effects opposite to the reaction time results, indicating that there was no speed-accuracy trade-off in this experiment.

### 2.4 Summary

The results of Experiment 1 indicated that when participants understand the third-person sentences of object as an inanimate object, ACE appears along the front-back axis. This suggests that when the subject of the action sentence is a personal name and the object is an inanimate object, participants simulate the action by considering themselves as the agent of the action. The direction of ACE aligns with the movement direction of the verb (Bergen and Wheeler, 2005). However, when the object is not an inanimate object, both person in the sentence could potentially act as agents. Therefore, in order to understand the relationship between the persons and the action, participants may simulate the person (agent or patient of action) in the left–right positions of space. In this case, the participant no longer simulates themselves as the agent/patient of the action, but rather as an observer of the action. We validated this in Experiment 2.

## 3 Experiment 2

### 3.1 Participants and materials

G*Power3.1 was used to calculate the required number of participants. Based on a medium effect size (*f* = 0.20), a statistical test power 1-*β* = 0.95, and a significance level of *α* = 0.05, the minimum required sample size was 112. A total of 124 college students (75 females) were recruited, aged 19–27 years old (*M* = 21.11, *SD* = 2.38), native Chinese, right-handed, normal visual acuity or corrected visual acuity. The subjects were randomly assigned equally to each of the four experimental conditions.

From the candidate materials, 20 sentences with third-person subjects and objects were selected, and the sentences describing action toward (e.g., 王强**拉回**了许文/Wang Qiang pulled Xu Wen back) or away from the body (e.g., 罗凯**推开**了程雪/Luo Kai pushed Cheng Xue away) were evenly divided. The two sets of sentences were matched for concreteness, validity, familiarity, emotional valence and stroke count. Additionally, 20 s-person sentences and 40 nonsensical sentences, similar to Experiment 1, were included.

### 3.2 Design and procedure

Same as Experiment 1.

### 3.3 Results

Six participants with accuracy lower than 85% were excluded, as well as extreme values with reaction times more than 2.5 standard deviations from the mean. The data exclusion rate was 4.49%. Descriptive statistics for mean accuracy and reaction time are presented in Table 2.

**Table 2:** Average reaction times and accuracy of sentences in experiment 2 (*M* ± *SD*).

Response axis	Reasonable response direction	RT (*ms*)		ACC (*%*)	
		STB	SAB	STB	SAB
Front-back axis	Toward the body (back)	1,302 ± 252	1,294 ± 247	94.22 ± 10.47	97.11 ± 4.85
	Away from the body (front)	1,318 ± 289	1,311 ± 316	96.89 ± 6.00	95.56 ± 13.37
Left–right axis	Toward the body (back)	1,107 ± 208	1,171 ± 175	98.81 ± 2.60	97.38 ± 3.32
	Away from the body (front)	1,277 ± 170	1,210 ± 165	98.44 ± 3.36	98.22 ± 4.61

STB, sentence describing actions toward the body; SAB, sentence describing actions away from the body.

The repeated measures ANOVA results for mean reaction time indicated a significant main effect of response axis [*F*_(1, 114)_ = 7.736, *p* = 0.006, $\eta_{p}^{2}$ = 0.064], with the left–right axis (*M* = 1,193, *SD* = 187) being faster than the front-back axis (*M* = 1,307, *SD* = 273). The main effect of reasonable response direction was not significant [*F*_(1, 114)_ = 2.142, *p* = 0.146], and the main effect of sentence types was not significant [*F*_(1, 114)_ = 0.147, *p* = 0.702].

The interaction between response axis and reasonable response direction was not significant [*F*_(1, 114)_ = 1.113, *p* = 0.294], nor was the interaction between response axis and sentence types [*F*_(1, 114)_ = 0.054, *p* = 0.816]. The interaction between reasonable response direction and sentence types was significant [*F*_(1, 114)_ = 7.308, *p* = 0.008, $\eta_{p}^{2}$ = 0.060].

The three-way interaction between response axis, reasonable response direction, and sentence types was significant [*F*_(1, 114)_ = 7.563, *p* = 0.007, $\eta_{p}^{2}$ = 0.062]. Analysis of simple effects revealed that, in the front-back axis direction, no significant difference in reaction time was observed between sentences describing action away from body when the key was pressed backward [*F*_(1, 114)_ = 0.112, *p* = 0.738] or forward [*F*_(1, 114)_ = 0.083, *p* = 0.774]. However, in the left–right axis, when the key was pressed to the left, the reaction time for sentences describing action toward the body was significantly faster than for sentences describing action away from the body (*F*_(1, 114)_ = 6.678, *p* = 0.011, $\eta_{p}^{2}$ = 0.055, 95% CI [14.901, 112.787]); when the key was pressed to the right, the reaction time for sentences describing actions away from the body was significantly faster than for sentences describing actions toward the body (*F*_(1, 114)_ = 7.992, *p* = 0.006, $\eta_{p}^{2}$ = 0.066, 95% CI [20.195, 114.762]).

The accuracy analysis results (see Supplementary materials) showed no main effects or interaction effects opposite to the reaction time results, indicating that there was no speed-accuracy trade-off in this experiment.

### 3.4 Summary

The results of this experiment showed that ACE only appeared in the left–right direction when participants understood action sentences with both the subject and object as third-person, similar to the results of van Dam and Desai (2017). Similarly, no ACE was found in the front-back axis in the experiments of Li (2015) and Schwarzkopf et al. (2011). This suggests that, when participants understood third-action sentences with both the subject and object as third-person, they mentally simulated the spatial perception, including the spatial relationship, with the agent on the left and the recipient on the right. The participants simulated the action from the observer’s perspective. Therefore, in Experiment 3, we aim to further examine whether activating the perspective of the agent/recipient along the front-back axis, which includes spatial information, would lead to the occurrence of ACE again.

## 4 Experiment 3

### 4.1 Participants and materials

G*Power3.1 was used to calculate the required number of participants. Based on a medium effect size (*f* = 0.20), a statistical power of 1 – *β* = 0.95, and a significance level of *α* = 0.05, the minimum required sample size was 56. Sixty-four college students (50 females), aged 19–27 years old (*M* = 20.92, *SD* = 2.34), native Chinese, right-handed, normal visual acuity or corrected visual acuity, were recruited and received a small amount of remuneration after the experiment. The participants were randomly divided equally between the two experimental conditions.

Based on the materials used in Experiment 2, 40 sentences were created by substituting different personal names, with 20 sentences describing actions toward the body and 20 sentences describing actions away from the body, ensuring that each verb appeared twice.

**Figure 2:**
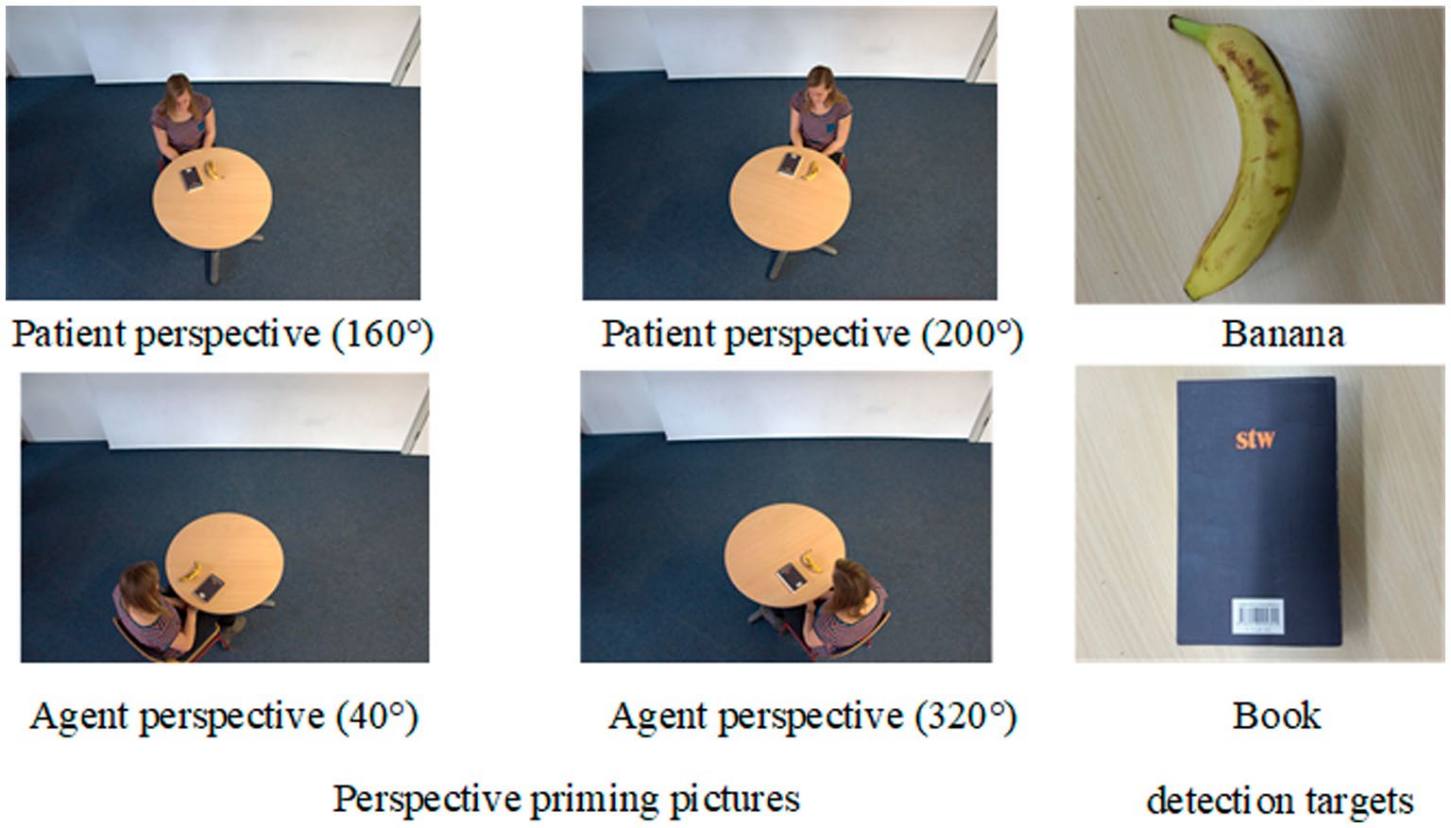
Perspective-taking materials used in Erle and Topolinski (2017). Reproduced with permission from the study of Erle and Topolinski (2017).

The perspective priming images were adapted from the materials used in Erle and Topolinski's (2017) study, as shown in Figure 2. In studies on spatial perspective-taking, the rotation angle of a person in an image influences participants’ choice of perspective. In a typical spatial perspective-taking task, participants are asked to determine which hand the depicted character is using to hold a target object (e.g., a book or flowers). Research has found that when the character’s rotation angle is <80° (e.g., 40° and 320° clockwise), reaction time is unaffected by the rotation angle, and participants make spatial judgments about the action from their own perspective, adopting an agent perspective where they themselves are positioned in the spatial background, and the character is in the foreground. However, when the rotation angle exceeds 80° (e.g., 160° and 200° clockwise), reaction time is influenced by the rotation angle, as participants mentally simulate the position of the character before making a spatial judgment. In this case, the character becomes the agent of the action, while the participant may adopt a patient perspective, with the character in the foreground and the participant in the background. Thus, the spatial perspective-taking task not only activates the participant’s relationship with the action but also activates the spatial relationship between the participant and others.

There are four types of character rotation angles: 40°, 160°, 200°, and 320° clockwise. Each angle includes two images, one featuring a male character and the other a female character, with books and bananas placed in their left and right hands. A book and a banana image were used as detection targets. The images with 40° and 320° rotations served as agent perspective priming images, where the participant’s left- and right-hand directions aligned with those of the character in the image. In contrast, the images with 160° and 200° rotations served as the patient perspective initiation images, where the participant’s left- and right-hand directions were mirror-image opposites of the characters.

### 4.2 Design and procedure

The experiment utilized a 2 (Perspective Type: agent perspective, patient perspective) × 2 [Reasonable response direction: toward the body (Back), away from the body (Front)] × 2 (Sentence types: describing action toward the body, describing action away from the body) mixed-design, with reasonable response direction as a between-subjects variable, and perspective type and sentence types as within-subjects variables. The dependent variables were the response time and accuracy in the sentence validity judgment task.

**Figure 3:**
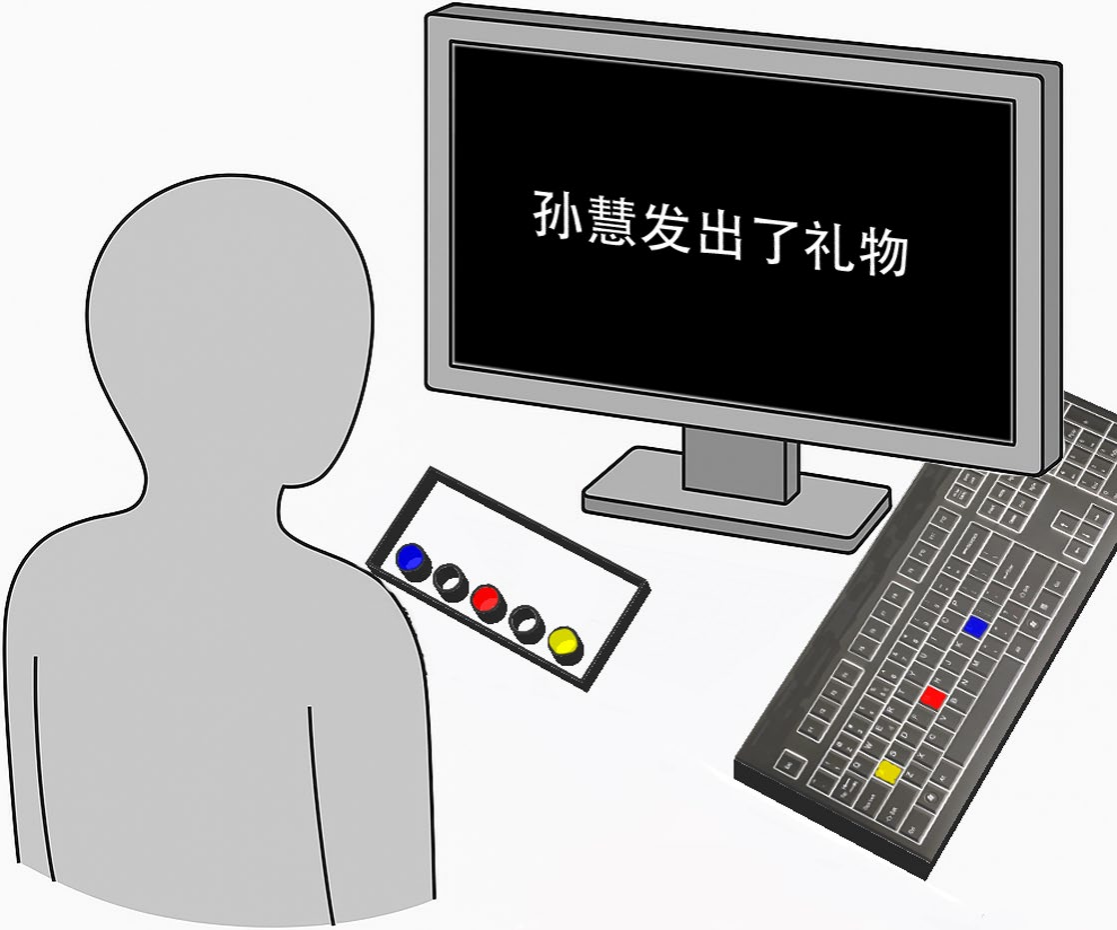
Experimental setup in experiment 3.

Building on Experiments 1 and 2, the SRBox response box from Psychology Software Tools was added as the response device for the perspective priming task (see Figure 3). In the perspective priming task, participants were required to judge which hand the character would use to hold the target object (e.g., a banana) from a specific perspective (e.g., the 40° agent-perspective priming). If the banana was on the left side of the character in the image, the participant needed to press the blue button on the left side of the response box (Button 1); if the banana was on the right side, the participant needed to press the yellow button on the right side of the response box (Button 5). After making a judgment, the participant would return their left index finger to the center red button (Button 3), preparing for the next judgment.

**Figure 4:**
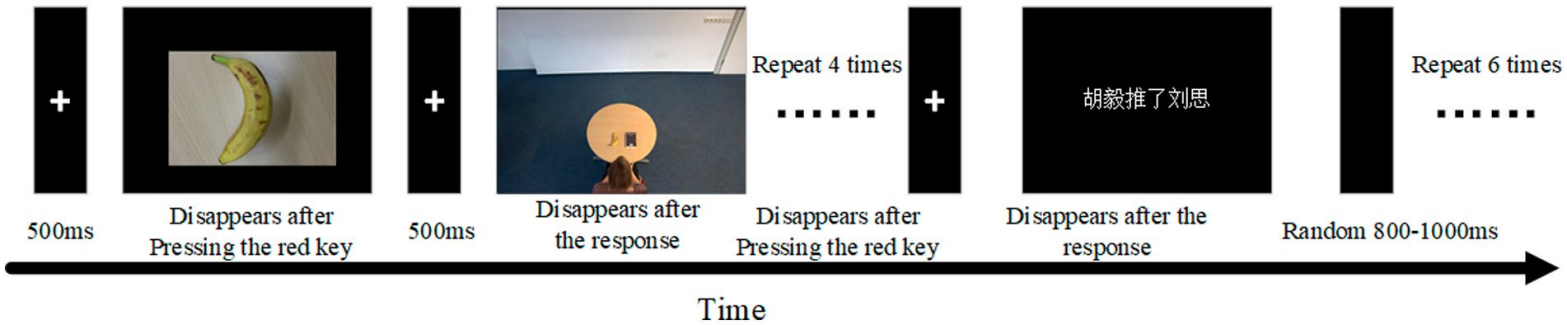
Procedure of experiment 3.

Following four perspective priming trials with their left hand on the response box, the participant would perform 6 sentence validity judgments with their right index finger on the keyboard, following the same key placement and requirements as in the previous experiments for the front-back axis conditions. In the sentence validity judgment task, an equal number of sentences were classified as plausible and implausible. Plausible sentences included 2 formal materials with the same action type and 1 filler sentence. This formed one cycle, as shown in Figure 4.

The two types of perspective priming and two types of the sentence constituted four experimental conditions, with 5 cycles per condition and 2 valid trials per cycle, for a total of 10 trials per condition. Each participant needed 25–30 min to complete the experiment.

### 4.3 Results

Four participants with accuracy below 85% were excluded. Cycles in which the number of correct responses in the 4 perspective priming tasks was fewer than 3 were removed, accounting for 1.75% of the total data. Extreme values outside of 2.5 standard deviations of the average response time were also excluded, representing 2.84% of the total data. The descriptive statistics for the participants’ average response time and accuracy are shown in Table 3.

**Table 3:** Average reaction times and accuracy of sentences in experiment 3 (*M* ± *SD*).

Perspective Type	Reasonable response direction	RT (*ms*)		ACC (*%*)	
		STB	SAB	STB	SAB
Agent perspective	Toward the body (back)	1,310 ± 203	1,310 ± 195	97.78 ± 3.64	94.00 ± 9.80
	Away from the body (front)	1,427 ± 197	1,333 ± 176	98.00 ± 4.32	95.78 ± 8.12
Patient perspective	Toward the body (back)	1,309 ± 215	1,243 ± 194	96.44 ± 6.72	95.56 ± 7.70
	Away from the body (front)	1,370 ± 182	1,361 ± 210	97.33 ± 7.75	96.67 ± 9.23

STB, sentence describing actions toward the body; SAB, sentence describing actions away from the body.

A repeated measures ANOVA on the average response time revealed that the main effect of reasonable response direction was not significant [*F*_(1, 58)_ = 3.355, *p* = 0.072]. Similarly, the main effect of perspective type was not significant [*F*_(1, 58)_ = 2.481, *p* = 0.121]. However, the main effect of sentence types was significant [*F*_(1, 58)_ = 7.005, *p* = 0.010, $\eta_{p}^{2}$ = 0.108]. Responses were faster for sentences describing actions toward the body (*M* = 1,312, *SD* = 196) compared to sentences describing actions away from the body (*M* = 1,354, *SD* = 202).

The interaction between response direction and perspective type was not significant [*F*_(1, 58)_ = 0.396, *p* = 0.532]. Similarly, the interaction between response direction and sentence type was not significant [*F*_(1, 58)_ = 0.334, *p* = 0.565], and the interaction between perspective type and sentence type was also not significant [*F*_(1, 58)_ = 0.109, *p* = 0.743].

The interaction between perspective types, reasonable response direction, and sentence types was significant [*F*_(1, 58)_ = 7.088, *p* = 0.010, $\eta_{p}^{2}$ = 0.109]. A simple main effects analysis revealed that under the agent perspective condition, when the key was pressed forward, sentences describing action away from body was significantly faster response time compared to sentences describing actions toward the body (*F*_(1, 58)_ = 10.842, *p* = 0.002, $\eta_{p}^{2}$ = 0.157, 95% CI [36.993, 151.706]). However, when the key was pressed backward, there was no significant difference between response times for sentence describing actions toward or away from the body [*F*_(1, 58)_ < 0.001, *p* = 0.991]. In contrast, under the patient perspective condition, no significant difference in response times was observed between sentence types when the key was pressed forward [*F*_(1, 58)_ = 0.091, *p* = 0.764]. Conversely, when the key was pressed backward, sentences describing actions directed away from the body elicited significantly faster response times than those describing actions toward the body (*F*_(1, 58)_ = 4.317, *p* = 0.042, $\eta_{p}^{2}$ = 0.069, 95% CI [2.431, 130.310]).

The accuracy analysis results (see Supplementary materials) showed no main effects or interaction effects opposite to the reaction time results, indicating that there was no speed-accuracy trade-off in this experiment.

### 4.4 Summary

Using sentence materials similar to those in Experiment 2, where the subject and object of the sentence are the personal name, an ACE along the front-back axis emerged when participants adopted the agent perspective. In this condition, participants simulated the action from the agent’s perspective. Conversely, when adopting the patient perspective, the ACE along the front-back axis reversed, with sentences describing away from the body facilitating key responses for toward the body. A similar reverse ACE has also been observed in other studies where the object of the sentence object was the pronoun “you,” explicitly designated the participant as the patient of the action (Glenberg and Kaschak, 2002; Schwarzkopf et al., 2011). The findings of this study indicate that by priming perspective, participants can also simulate themselves as the agent or patient of the action, rather than adopting the observer’s perspective for action simulation.

## 5 General discussion

The results of Experiment 1 and Experiment 2 suggest that the animacy of nouns influences the spatial axis preference when readers mentally simulate a sentence, thus affecting the direction of action simulation in the sentence. Animate nouns are more likely to serve as the subject and the agent, while inanimate nouns tend to serve as the object and the patient (Comrie, 1989; Mak et al., 2002; Trueswell et al., 1994). When both the subject and object in a sentence are personal names, ACE is observed only along the left–right axis. The inconsistencies in previous studies may be attributed to variations in the animacy of the object (Li, 2015; Bergen and Wheeler, 2005; Schwarzkopf et al., 2011).

According to the perceptual symbol systems theory, understanding action sentences involves simulating the perceptual-motor scenario constructed by the entire sentence (Barsalou, 1999). Research has shown that even a brief reading experience (e.g., a square between a cross and a triangle) is enough to construct a consistent and specific spatial simulation (Román et al., 2015), and body-related verbs in Chinese single characters can evoke spatial simulations (Wang et al., 2019). In general, action simulation integrates the verb’s action with other components of sentence, forming a simulation of movement and perception within the overall spatial framework (Bidet-Ildei et al., 2020; Fischer and Zwaan, 2008).

For sentences with third-person and the object is an inanimate object, readers default to adopting the agent’s perspective, resulting in ACE along the front-back axis. When both the subject and object as personal name and perspective information is unclear, participants tend to adopt the observer’s perspective, leading to ACE along the left–right axis. Researchers suggest that the spatial simulation along the left–right axis are related to the left hemisphere processing advantage for language and the direction of writing. For example, Maass and Russo (2003) found that Italian participants, who write from left to right, tend to order agents and patients in sentences with third-person subjects and objects from left to right, while Arabic participants, who write from right to left, tend to order them from right to left. Thus, changes in the animacy of the object can alter the direction of ACE. Once a clear perspective is activated through front-back axis response directions, readers will subsequently adopt either the agent’s or patient’s perspective.

Experiment 3 further demonstrates that perspective activation, which includes spatial information, influences the sentence comprehension process. Perceptual perspectives and the spatial framework they simulate not only influence cognitive processes like social reasoning (Erle and Topolinski, 2017; Xie et al., 2018) but also affect language comprehension. The ACE results indicate that the spatial perspective priming task can influence the perspective adopted in subsequent language comprehension. Meanwhile, the reversed ACE suggests that changes in the spatial position of mental simulation can influence the direction of ACE, a finding similar to previous research (Gianelli et al., 2011). Gianelli et al. (2011) found that variations in the left–right spatial position of the activated agent/patient also affect the direction of ACE along the front-back axis. In addition, these results suggest that although some participants may have a preference for a certain perspective during language comprehension (Vukovic and Williams, 2015), they can still flexibly shift perspectives based on activation cues. Moreover, beyond motion and spatial perception simulation, language understanding involves other types of perceptual simulations. For example, when comprehending moral concepts, readers can map space (Dong et al., 2018) or relate to perceptual aspects such as colors (e.g., black-and-white) or body cleanliness (Ding and Wang, 2019). Language comprehension is a process of multimodal simulation (Barsalou, 1999; Bidet-Ildei et al., 2020). These findings support the perceptual symbol systems theory, suggesting that participants can flexibly simulate action perspectives based on spatial cues when processing third-person action sentences.

However, the experimental results show that ACE is instable and sometimes appears in only one direction. Recent studies have revealed the instability of ACE (Morey et al., 2022; Jin et al., 2024), which may be attributed to several factors. Firstly, it may be influenced by participants’ attention to different information in a sentence. Studies have shown that ACE varies with changes in font and underlines used to emphasize different components of a sentence, and the matching effect only emerges when the verb is the emphasized information (Jin et al., 2024). Secondly, this instability may also stem from individual differences in perspective preference, with some participants tending to adopt a fixed perspective during the experiment. Since participants’ perspectives in language comprehension are not necessarily influenced by pronouns (Vukovic and Williams, 2015), future research could consider perspective preference as a controlled variable. Furthermore, to ensure consistency across various linguistic variables, the number of materials used in this study was relatively limited. Future research could expand the materials or employ natural corpora for further exploration. Additionally, using different paradigms and parameters could provide a conceptual extension to examine ACE more thoroughly (Zwaan, 2021).

## 6 Conclusion

Based on the conditions of this study, the following conclusions can be drawn:

1 The direction of ACE in comprehending third-person action sentences is influenced by the animacy of the object. When the object is an inanimate noun, ACE manifests along the front-back axis; when the object is a personal name, ACE appears along the left–right axis.
2 Perspective priming affects the direction of ACE during the comprehension of third-person action sentences. Specifically, the agent/patient perspective primed by the image induced the participants to adopt the agent/patient perspective while understanding the sentences, resulting in opposite ACE along the front-back axis.
3 Readers can flexibly simulate action perspectives based on spatial information, which supports the perceptual symbol systems theory.

## Data Availability

The raw data supporting the conclusions of this article will be made available by the authors, without undue reservation.
