# Supplementary material for: The action-sentence compatibility effect in third person action sentence comprehension
Source: Front Psychol. 2025 Jun 4;16:1562351. doi: 10.3389/fpsyg.2025.1562351 (PMC12175436; doi:10.3389/fpsyg.2025.1562351)
Supplement: Supplementary file 1 [file Table_1.docx]

Supplementary Material

**Experiment 1 Accuracy rate analysis**

The results of the repeated measures ANOVA on mean response accuracy rate shown that the main effect of response axis was not significant (*F*(1, 116) = 0.237, *p* = 0.627), the main effect of reasonable response direction was not significant (*F*(1, 116) = 0.237, *p* = 0.627), and the main effect of sentence types was not significant (*F*(1, 116) = 2.207, *p* = 0.140).

The interaction between response axis and reasonable response direction was significant (*F*(1, 116) = 10.337, *p* = 0.002). On the front-back axis, the accuracy for the Away from the body(front) condition (*M* = 0.963, *MSE* = 0.006) was higher than for the Toward the body(back) condition (*M* = 0.963, *MSE* = 0.006). On the left-right axis, the accuracy for the Toward the body(left) condition (*M* = 0.984, *MSE* = 0.006, $\eta_{p}^{2}$ = 0.082) was higher than for the Away from the body(right) condition (*M* = 0.963, *MSE* = 0.006). The interaction between response axis and sentence types was not significant (*F*(1, 116) = 0.153, *p* = 0.697), and the interaction between reasonable response direction and sentence types was not significant (*F*(1, 116) = 0.006, *p* = 0.938).

The three-way interaction between response axis, correct response direction, and sentence type was significant (*F*(1, 116) = 2.696, *p* = 0.103).

**Experiment 2 Accuracy rate analysis**

The results of the repeated measures ANOVA on mean response accuracy rate shown that the main effect of response axis was significant (*F*(1, 116) = 6.083, *p* = 0.015), and the accuracy on the left-right axis (*M* = 0.982, *MSE* = 0.007) was higher than on the front-back axis (*M* = 0.959, *MSE* = 0.006). the main effect of reasonable response direction was not significant (*F*(1, 116) = 0.186, *p* = 0.667), and the main effect of sentence types was not significant (*F*(1, 116) = 0.001, *p* = 0.980).

The interaction between response axis and reasonable response direction was no significant (*F*(1, 116) = 0.030, *p* = 0.863), the interaction between response axis and sentence types was not significant (*F*(1, 116) = 0.742, *p* = 0.391), and the interaction between reasonable response direction and sentence types was not significant (*F*(1, 116) = 0.657, *p* = 0.419).

The three-way interaction between response axis, correct response direction, and sentence type was significant (*F*(1, 116) = 2.127, *p* = 0.147).

**Experiment 3 Accuracy rate analysis**

A repeated measures ANOVA on the average accuracy rate revealed that the main effect of reasonable response direction was not significant (*F*(1, 58) = 1.291, *p* = 0.261). Similarly, the main effect of perspective type was not significant, *F*(1, 58) = 0.039, *p* = 0.844, and the main effect of sentence types was no significant, *F*(1, 58) = 3.278, *p* = 0.075.

The interaction between response direction and perspective type was not significant, *F*(1, 58) = 3.895, *p* = 0.053. Similarly, the interaction between response direction and sentence type was not significant, *F*(1, 58) = 0.648, *p* = 0.424, and the interaction between perspective type and sentence type was also not significant, *F*(1, 58) < 0.001, *p* > 0.999.

The interaction between perspective types, reasonable response direction, and sentence types was significant (*F*(1, 58) = 0.357, *p* = 0.552).
